# Mechanosensitive molecular mechanisms of myocardial fibrosis in living myocardial slices

**DOI:** 10.1002/ehf2.13832

**Published:** 2022-02-06

**Authors:** Raquel Nunez‐Toldra, Thomas Kirwin, Elisa Ferraro, Fotios G. Pitoulis, Laura Nicastro, Ifigeneia Bardi, Worrapong Kit‐Anan, Julia Gorelik, Andre R. Simon, Cesare M. Terracciano

**Affiliations:** ^1^ National Heart and Lung Institute Imperial College London London UK; ^2^ Royal Brompton and Harefield NHS Foundation Trust London UK

**Keywords:** Myocardial slices, Mechanical load, Myocardial fibrosis, Heart failure

## Abstract

**Aims:**

Altered mechanical load in response to injury is a main driver of myocardial interstitial fibrosis. No current *in vitro* model can precisely modulate mechanical load in a multicellular environment while maintaining physiological behaviour. Living myocardial slices (LMS) are a 300 μm‐thick cardiac preparation with preserved physiological structure and function. Here we apply varying degrees of mechanical preload to rat and human LMS to evaluate early cellular, molecular, and functionality changes related to myocardial fibrosis.

**Methods and results:**

Left ventricular LMS were obtained from Sprague Dawley rat hearts and human cardiac samples from healthy and failing (dilated cardiomyopathy) hearts. LMS were mounted on custom stretchers and two degrees of diastolic load were applied: physiological sarcomere length (SL) (SL = 2.2 μm) and overload (SL = 2.4 μm). LMS were maintained for 48 h under electrical stimulation in circulating, oxygenated media at 37°C. In overloaded conditions, LMS displayed an increase in nucleus translocation of Yes‐associated protein (YAP) and an up‐regulation of mechanotransduction markers without loss in cell viability. Expression of fibrotic and inflammatory markers, as well as Collagen I deposition were also observed. Functionally, overloaded LMS displayed lower contractility (7.48 ± 3.07 mN mm^−2^ at 2.2 SL vs. 3.53 ± 1.80 mN mm^−2^ at 2.4 SL). The addition of the profibrotic protein interleukin‐11 (IL‐11) showed similar results to the application of overload with enhanced fibrosis (8% more of collagen surface coverage) and reduced LMS contractility at physiological load. Conversely, treatment with the Transforming growth factor β receptor (TGF‐βR) blocker SB‐431542, showed down‐regulation of genes associated with mechanical stress, prevention of fibrotic response and improvement in cardiac function despite overload (from 2.40 ± 0.8 mN mm^−2^ to 4.60 ± 1.08 mN mm^−2^).

**Conclusions:**

The LMS have a consistent fibrotic remodelling response to pathological load, which can be modulated by a TGF‐βR blocker. The LMS platform allows the study of mechanosensitive molecular mechanisms of myocardial fibrosis and can lead to the development of novel therapeutic strategies.

## Introduction

Cardiac injury results in pathological structural remodelling of the myocardium including the development of myocardial fibrosis. This leads to impaired relaxation with the development of diastolic dysfunction and heart failure (HF) in some patients.^1^ With HF now affecting 2% of the global population, there is an urgent need for effective anti‐fibrotic therapies.^2^ Cardiac fibroblasts (CFs) are Collagen‐producing mesenchymal cells, comprising 15% of the total cardiac cellular composition.^3^ They play a crucial role in extracellular matrix homeostasis, as well as contributing to the structural, mechanical, and electrical properties of the myocardium.^4^ CFs produce and turn over collagenous extracellular matrix as part of the normal adaptive response to increased mechanical load of the heart. However, chronic overload because of hypertension or myocardial injury triggers a repair program that culminates in the transformation of CFs into myofibroblasts. These are characterized by the expression of contractile proteins, collagen secretion and pro‐inflammatory and pro‐fibrotic cytokine release, and are the main drivers for the development of fibrosis.^1^, ^5^ The activation of CFs in response to mechanical stress relies on their mechanosensory ability. Their phenotype is determined by the stretch and stiffness of the tissue in which they lie. This adaptive response involves mechanotransduction signalling pathways, whereby external stiffness regulates the cytoskeleton.^6^

Studying the fibrotic response to mechanical load *in vitro* is hampered by the lack of appropriate experimental models that successfully imitate the *in vivo* process. The study of CFs in intact hearts does not allow direct observation of time‐dependent changes and is too complex to determine cell origin and regulation. Culture of isolated CFs on plastic culture dishes or polydimethylsiloxane membranes used to impose cyclic stretch induce rapid profibrotic myofibroblast activation, independent of mechanical or humoral factors.^7^ These materials have stiffnesses five and three fold, respectively, greater than that of cardiac muscle.^8^ The spontaneous transdifferentiation of CFs into myofibroblasts has been prevented by the introduction of hydrogels with low stiffness. However, while 3D hydrogel‐platforms permit a heterocellular environment, the native architecture of the myocardium is still lost. Additionally, cardiomyocytes incorporated in hydrogels (usually neonatal or derived from pluripotent stem cells), do not reach enough maturity to represent adult cardiomyocytes limiting the relevance of these studies.^6^ Furthermore, *in vitro* 3D models cannot accurately predict the effect on cardiac function as they often use image‐based analysis to quantify contractility, rather than strain‐based measurements of force.^4^, ^9^ Therefore, no *in vitro* model can precisely modulate mechanical load in a multicellular environment, while also maintaining the physiological behaviour of CFs.

Living myocardial slices (LMS) are ultrathin (300 μm) preparations of ventricular myocardium that retain the cardiac cellular composition, architecture and physiology of the heart.^10^, ^11^ When cultured *in vitro*, they still display viability and their physiology is maintained for several days after preparation.^12^, ^13^ The multicellularity, the 3D nature and the presence of viable myocardium in culture allow CFs to maintain physiological interactions with the other cardiac cells and the extracellular matrix (ECM).^14^ ^.^ ^15^In this study, we exploited the ability of rat and human LMS to respond to cardiac remodelling differently depending on physiological (SL = 2.2 μm) and pathological preload (SL = 2.4 μm, overload). Additionally, we compared the effect of mechanical load with the administration of interleukin‐11 (IL‐11), a crucial fibroblast‐specific factor involved in myocardial fibrosis.^16^ Finally, a selective TGF‐βR blocker, a potential anti‐fibrotic drug, was tested on the LMS model to evaluate the reversibility of the load‐induced fibrotic phenotype and the potential of LMS for anti‐fibrotic drug discovery.

## Methods

### Living myocardial slices preparation

The investigation conforms to the Guide for the Care and Use of Laboratory Animals published by the US National Institutes of Health (NIH Publication No. 85‐23, revised 1985) and to the principles outlined in the *Declaration of Helsinki* (*Br Med J* 1964**; ii:** 177).

All animal experiments complied with institutional and national regulations, and approved by Imperial College London, under licence by the UK Home Office, United Kingdom Animals (Scientific Procedures) Act 1986, Amendment Regulations 2012, and EU directive 2010/63/EU. Human samples were provided by the NIHR Cardiovascular Biomedical Research Unit at the Royal Brompton and Harefield NHS Foundation Trust and Imperial College London. The study performed was approved by a UK institutional ethics committee (NRES ethics number for biobank samples: 09/H0504/104 + 5; Biobank approval number: NP001‐06‐2015 and MED_CT_17_079) and Imperial College London. Informed consent was obtained from each patient/family involved in this study.

LMS were prepared and cultured as previously described.^12^ Sprague–Dawley rats (300–350 g) were sacrificed under isoflurane‐induced anaesthesia (4% isoflurane at 4 L/min oxygen) by cervical dislocation. The heart was quickly harvested and placed in cold Tyrode's slice solution (30.0 mM 2, 3‐butanedione monoxime, 140.0 mM NaCl, 9.0 mM KCl, 10 mM glucose, 10.0 mM HEPES, 1.0 mM MgCl_2_, 1.0 mM CaCl_2_ at pH of 7.40). The left ventricle was isolated from the rest of the heart and extra‐heart tissues, opened by an incision down the interventricular septum and flattened via incisions to the papillary muscles. The tissue was mounted on a specimen holder coated in 4% agarose, epicardial side down, using surgical glue (Histoacryl, Braun). The specimen holder was placed in a vibratome bath filled with cold Tyrode's slicing solution, superfused with 100% O_2._ Using a high precision vibratome (7000 amz‐2, Campden Instruments) with a ceramic blade, the tissue was sliced (300 μm thickness) longitudinal to the fibre orientation from the endocardium down.

Human hearts (Supporting information *Table* *S1*) were perfused with cold cardioplegia solution and arrested *in situ* prior to being explanted. The specimens were placed in cold cardioplegia, placed on ice and transported to the laboratory. The left ventricle of the specimen was identified and isolated from the rest of the tissue. A 1.5 cm^2^ tissue block was dissected out of the left ventricular free wall by making an incision through the full thickness of the ventricular wall. The tissue was mounted epicardial surface down and sliced in the same manner as rat LMS.

### Living myocardial slices culture

**Figure 1:**
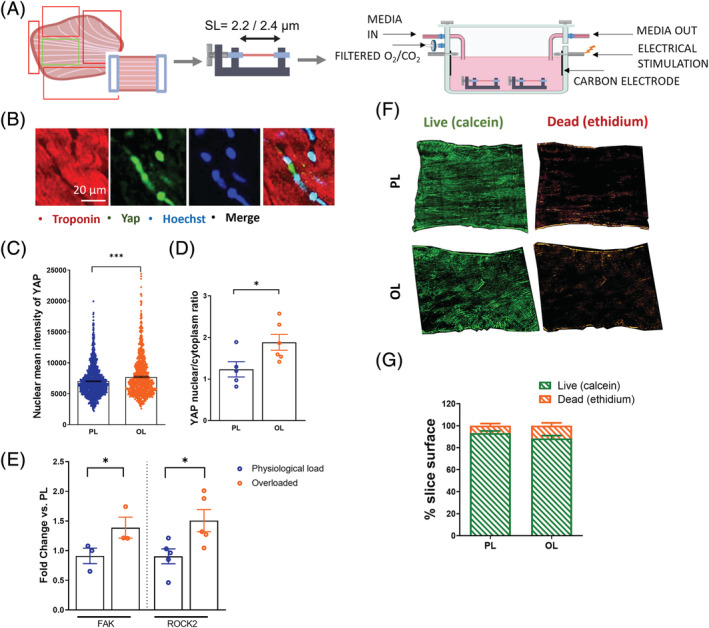
Electro‐mechanical culture of living myocardial slices (LMS). (A) Diagram of the experimental design. (B) Representative images of rat LMS showing YAP nuclear and cytoplasm expression. (C) YAP mean nuclear intensity after 48 h of culture (*N* = 5). (D) YAP nuclear/cytoplasm ratio after 48 h of culture (*N* = 5). (E) Fold change expression of genes activated by mechanical stress, FAK (*N* = 3) and ROCK2 after 48 h of culture (*N* = 5). (F) Cell viability analysed by live/dead staining. (G) Area quantification of living (Calcein) and dead (ethidium) cells on myocardial slices after 48 h of culture (*N* = 3). *P* value was calculated using a Student's *t* test. *, **, *** = *P* values <0.05, 0.01, and 0.001, respectively. Data shown as mean ± SEM. PL, physiological load; OL, overload; YAP, yes‐associated protein.

Once the slice was generated, fibre alignment was visualized under light microscopy and an aligned area dissected. The length and width of the slice were measured for normalization of force to the cross‐sectional area. Custom‐made biocompatible 3D‐printed T‐Glase rectangular holders were attached perpendicular to the fibres along the width of the slice using surgical glue. The slice was placed on the stretcher and extended according to the pre‐load condition. Watson et al used laser diffraction to establish the % stretch (distance between stretcher posts/resting length of myocardial slice x 100) required to produce a SL (μm) that correlated with a mechanical preload condition (*Table* *1*). Four slices were placed in each chamber and superfused with oxygenated media. The culture media (Medium‐199 with Earl's salts; Sigma‐Aldrich, UK) was prepared by adding 0.1% ITS (Sigma‐Aldrich, UK) and 2% penicillin/streptomycin (Sigma‐Aldrich, UK). Hormones (adrenaline 4 nM; noradrenaline 4 nM dexamethasone 100 nM, and triiodothyronine (T3) 2.15 nM) with the addition of ascorbic acid (20 μg/ml) (all from Sigma‐Aldrich, UK) were also added to the media to maintain the physiological properties of the LMS during culture. Sealed chambers were placed in an incubator at 37°C and cultured for 48 h, and the media was constantly circulated through a peristaltic pump at 15 mL/min. Field stimulation was delivered via carbon electrodes at 1 Hz (width 10 ms, 15 V) for rat slices and 0.5 Hz (width 10 ms, 15 V) for human slices. At 24 h, 30 mL of media was added to the chambers to account for the media lost due to evaporation (*Figure* *1A*).

**Table 1:** Stretch of LMS required to recapitulate the pre‐load conditions in culture based on previous data which used laser diffraction to establish the SL that characterizes each condition and the % stretch of the slice required to reach these SL^12^

Sample	Physiological stretch (SL = 2.2 μm)	Overloaded stretch (SL = 2.4 μm)
Rat LMS	17.5%	27.3%
Human HF LMS	19.6%	29%
Human non‐HF LMS	23%	34%

HF, heart failure; LMS, living myocardial slices; SL, sarcomere length.

For the IL‐11 cultures, rat recombinant IL‐11 at 10 nM (Cloud‐clone, USA) was added to the media. TGF‐βR blocker experiments, SB‐431542 (Tocris, UK), was dissolved in DMSO (Sigma‐Aldrich, UK) and added to the chamber to reach a final concentration of 3 μM. The control was a chamber with DMSO at equivalent volume to that added with the drugs.

The LMS contractility measurements, and CFs isolation were performed immediately after culture. Following measurement of maximum contractility, LMS for histology and molecular analyses were quickly washed with phosphate‐buffered solution (PBS) and either fixed or snap‐frozen in liquid nitrogen and stored at −80°C.

### Contractility

The LMS contractility was assessed using a force transducer (Harvard Apparatus, USA). To assess contractility cultured LMS were removed from the culture chamber and the stretchers and transferred to the force transducer using the attached holders. Rat myocardial slices were field stimulated at 1 Hz, 10 ms, and 20–30 V. Human HF and non‐HF slices were field stimulated at 0.5 Hz, 10 ms, and 20–30 V. LMS were continuously superfused in 37°C oxygenated Tyrode's solution and progressively stretched in a stepwise manner, until maximum isometric contraction was obtained. Data were recorded using AxoScope software, and peak amplitude analyses were conducted using Clampfit software (both Molecular Devices, USA).

### Fibroblast isolation

For fibroblasts isolation LMS were digested immediately after culture with collagenase‐A for 20 min two times (Roche, 0.229 U/mg) dissolved in Tyrode's solution. The digested tissue was minced, and 5% foetal bovine serum was added to block collagenase. The cell suspension was then filtered to remove cell debris and centrifuged at 14 000 rpm. Rat Cardiac Fibroblast Isolation Kit (Milteny Biotec, UK) and a MACS separator were used to isolate CFs (*Figure* *S1A*). An immunostaining of non‐fibroblasts and fibroblasts markers was performed in order to confirm the cell isolation methods (*Figure* *S1B*).

### Gene expression

The relative expression of the fibrotic and inflammatory markers was studied by quantitative real‐time reverse transcription‐polymerase chain reaction (qRT‐PCR). Following measurement of maximum contractility, LMS were quickly washed with PBS and holders were dissected off with a razor blade. LMS were then immediately submerged in liquid nitrogen and placed in a −80°C freezer until mRNA isolation.

For mRNA extraction, frozen LMS were transferred to a pre‐cooled 2 mL round‐bottom tube containing a 5 mm stainless‐steel ball and 500 μL of TRIzol (Thermo Fisher Scientific, UK) and placed in a TissueLyser LT (Quiagen) at 40 Hz and ran for 3–5 min until the sample was completely lysed. A 100 μL of chloroform (Sigma‐Aldrich, UK) was added to each tube. The tube was then left in a rack for 5 min at room temperature and intermittently shaken. Then, it was centrifuged at 12 000 rpm for 15 min and phase separation occurred. The top aqueous layer (containing the RNA) was transferred to a new tube and an equivalent volume of 70% ethanol was then added and mixed. The solution was finally transferred to an RNeasy spin column (Qiagen, UK) and the manufacturer's instructions were then followed.

A5 00 ng of the isolated mRNA was reverse transcribed to cDNA with ISCRIPT Reverse Transcriptase Kit (Bio‐Rad, UK) following the manufacturer's protocol. The qPCR mixture contained 37 ng cDNA, 300 nM of forward and reverse primers (*Table S2*) and iTaq SYBR Green Supermix (Bio‐Rad, UK). Finally, amplifications were performed in an Eppendorf RealPlex Real‐time PCR system. The data were normalized to the expression of the housekeeping gene GAPDH and the mean expression of 2.2 SL condition.

### Live/dead viability assay

Following culture, LMS were removed from the stretchers and the rings were detached using a razor blade. The stock reagents were diluted following manufacturer's instructions to produce a solution with 2 μM calcein‐AM and 4 μM ethidium homodimer‐1 (Molecular probes, Thermofisher). 2 mL of solution was added to each well of a six‐well plate and slices were placed in the individual wells and incubated at 37°C for 45 min in a humidified, 5% CO_2_ atmosphere. Following incubation, slices were washed in PBS and then fixed in 4% formaldehyde solution for 15 min at room temperature. Tiled images of the whole myocardial slice were automatically collected using a ×10 objective on a Zeiss AxioObserver equipped with a motorized stage. To assess slice surface viability, we measured the percentage of area stained with fluorescent calcein‐AM and ethidium homodimer‐1 signal using ImageJ software.

### Immunofluorescence staining

Following culture, LMS were washed in PBS and then fixed in 4% formaldehyde solution for 15 min at room temperature. LMS were permeabilized in 1% Triton X‐100 and blocked in 10% foetal bovine serum, 5% bovine serum albumin and 10% horse serum in PBS solution for 3 h at room temperature. LMS were incubated with the corresponding primary antibody αSMA (M0851 mouse monoclonal antibody, Dako, 1:1000), Collagen I (ab34710 rabbit polyclonal antibody, Abcam, 1:1000), Vimentin (pa1‐10003 chicken, Invitrogen, 1:4000), Cardiac troponin (ma5‐12960, Thermofisher, 1:1000), YAP1 (sc‐101199, Santa Cruz, 1:100) in PBS at 4°C overnight, and then washed three times for 30 min. LMS were incubated in the secondary antibody in PBS for 2 h at room temperature. LMS were then washed three times for 30 min, stained with Hoechst (Thermofisher, 1:1000) during 15 min, washed again three times for 15 min and stored in PBS at 4°C. Immunolabelled slices were imaged using a confocal microscope (Zeiss LSM‐870).

### Protein extraction and Western blots

Protein expression of the fibrotic and inflammatory markers was studied by Western blot. Following measurement of maximum contractility, LMS were quickly washed with PBS and the rings were dissected off with a razor blade. The LMS was then immediately snap‐frozen in liquid nitrogen and stored at −80°C for analysis. Total protein samples were prepared as previously described.^17^ LMS were crushed in a fine powder (20 mg) using a pestle and mortar under liquid nitrogen, suspended in 0.1 mg/μL SB20 solution and sonicated for approximately 1 min. After the addition of 2‐mercaptoethanol (2.5% final concentration), proteins (50 μg) were fractionated on 8% sodium dodecyl sulfate‐polyacrylamide gel electrophoresis (SDS‐PAGE), electrophoretically transferred to 0.2 μM polyvinylidene difluoride (PVDF) immunoblot membranes (BIO‐RAD) and probed for Gapdh (PA1‐987 rabbit polyclonal antibody, Invitrogen, 1:2000), αSMA (M0851 mouse monoclonal antibody, Dako, 1:1000), Collagen I (ab34710 rabbit polyclonal antibody, Abcam, 1:1000) and CD45 (ab10558 rabbit polyclonal, Abcam, 1:500) overnight at 4°C. Bands of interest were detected using Alexa Fluor 488 donkey anti‐mouse IgG and Alexa Fluor 546 anti‐rabbit IgG (1:2000), following an incubation time of 3 h at room temperature. Blots were scanned and quantitative analysis was performed using ImageJ software.

### Statistical analysis

Data analysis was performed using Prism 8 software (GraphPad, USA). All data are expressed as standard error mean (SEM). Statistical analysis was performed using Student's *t* test, one‐way or two‐way ANOVA with Bonferroni *post hoc* test as applicable. *N* = number of biological replicates. Significance was defined as *P* < 0.05.

## Results

### Living myocardial slices for the study of mechanosensitive molecular mechanisms of myocardial fibrosis

LMS were stretched uniaxially to either 2.2 μm SL (physiological condition) or 2.4 μm SL (overloaded condition) and cultured for 48 h.^12^ Culture media was oxygenated directly in the chamber and field stimulation continuously provided via carbon electrodes (*Figure* *1A*). Following 48‐h culture, the effect of mechanical load on YAP nuclear staining (*Figure* *1B–D*) and the expression of genes activated by mechanical stress (*Figure* *1E*) were measured. Rat LMS cultured in overloaded conditions showed an increase in mean YAP nuclear intensity and a higher YAP nuclear/cytoplasm ratio. FAK and ROCK2 were also up‐regulated in overloaded LMS. A live/dead assay was performed to determine if the increase in mechanical preload affected LMS viability and no significant differences between the viability of LMS cultured at either mechanical load were found (*Figure* *1F–G*).

**Figure 2:**
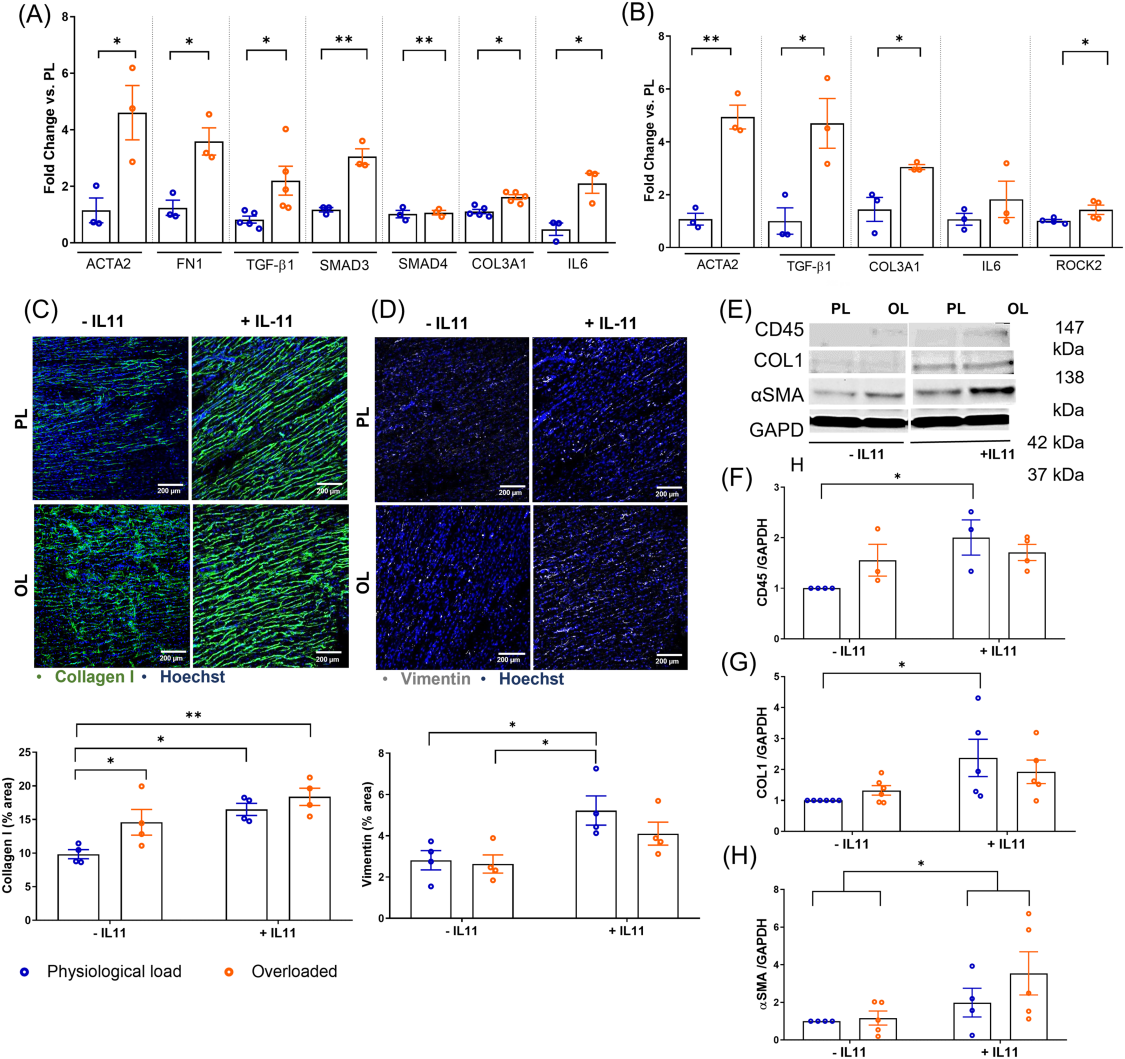
Rat living myocardial slices (LMS) for the study of mechanosensitive molecular mechanisms of myocardial fibrosis. (A) Fold change expression of pro‐fibrotic genes *ACTA2*, *FN1*, *TGF‐β1*, *SMAD3*, *SMAD4*, Collagen production gene *COL3A1*, and pro‐inflammatory gene IL‐6 in myocardial slices after 48 h of culture (*N* = 3–5). (B) Fold change expression of pro‐fibrotic, pro‐inflammatory and genes activated by mechanical load in fibroblasts isolated from 48 h cultured LMS (*N* = 9, each sample contains 3 LMS). (C) Representative images of rat LMS stained for Collagen I/Hoechst and (D) Vimentin/Hoechst after 48 h of culture with and without IL‐11 (*N* = 4). (E) Percentage quantifications of the LMS area covered by Collagen I and Vimentin. (F–I) Example images and quantifications of Western blots of CD45, COL1, αSMA, and GAPDH from 48 h cultured myocardial slices with and without IL‐11 (*N* = 4). *P* value was calculated using a Student's t test (gene expression) or two‐way ANOVA with Bonferroni's multiple comparison (IL‐11 experiments). *, **, ***, **** = *P* value <0.05, 0.01, 0.001, and 0.0001, respectively. Data shown as mean ± SEM. PL, physiological load; OL, overload.

To assess whether biomechanical stress can activate ECM remodelling in LMS, we measured fibrosis‐related gene expression profiles by RT‐qPCR analysis of mRNA isolated from 48 h cultured slices (*Figure* *2A*) and from isolated CFs (*Figure* *2B*). Compared with physiological load, overloaded LMS had increased expression of profibrotic genes such as smooth muscle alpha actin 2 (ACTA2), Fibronectin 1 (FN1) and TGF‐β1. Members downstream SMAD pathway were also analysed. While SMAD3 expression was markedly increased, there were no differences in SMAD4 expression, suggesting that biomechanical stress differentially regulates SMAD subtypes (*Figure* *2A*). Overloaded slices showed a higher gene expression of interleukin 6 (IL‐6), indicating a possible involvement of a local inflammatory response. Furthermore, overloaded slices had a higher expression of Collagen Type III (COL3A1), which correlated with an increase in Collagen deposition demonstrated by immunofluorescence staining (*Figure* *2C*). However, there were no differences in Vimentin expression indicating that there was no effect on stromal cell proliferation (*Figure* *2D*).

To specifically assess CFs differentiation into myofibroblasts without confounding factors due to the presence of other cell types, we isolated CFs from 48 h cultured LMS (*Figure* *2B*, *Figure* *S1*). Isolated CFs showed a similar gene expression pattern to whole LMS with more differences in pro‐fibrotic or ECM remodelling genes such as ACTA2, TGF‐β1, or COL3A1 in overloaded LMS but without differences in pro‐inflammatory genes such as IL‐6, suggesting a more fibrotic than inflammatory role in CFs. There was also an increase in ROCK2 expression in the overloaded CFs, possibly activated by mechanical stress from the YAP/TAZ pathway.

In order to compare our findings with a load‐independent cause of fibrosis, the pro‐fibrotic factor IL‐11^16^ was added to the culture media. 10 nM IL‐11 enhanced the fibrotic phenotype of the LMS particularly at physiological sarcomere length condition. Collagen I secretion (*Figure* *2C*), Collagen I (COL1) protein expression (*Figure* *2E*, *2G*), stromal cell proliferation (Vimentin expression) (*Figure* *2D*), and CD45 (*Figure* *2E*, *2F*) were up‐regulated by the presence of IL‐11 at physiological load condition. αSMA expression was increased by IL‐11 without significant differences between loads (*Figure* *2E*, *2H*).

**Figure 3:**
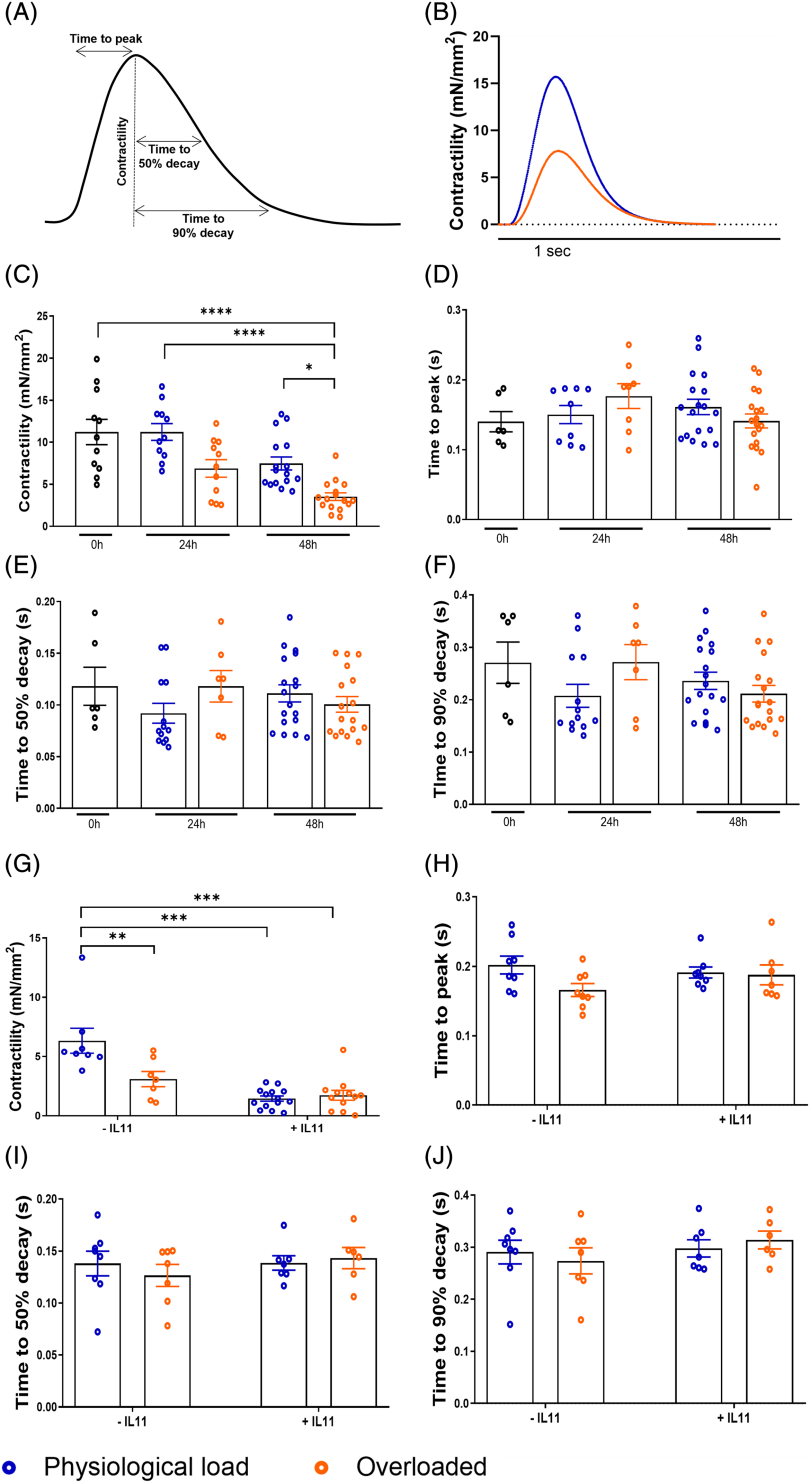
Functional remodelling of rat living myocardial slices (LMS). (A) Representative force trace showing different parameters. (B) Representative traces of rat LMS contraction at 2.2 μm SL and 2.4 μm SL after 48 h of culture. (c) Contractility of LMS as measured by normalising the amplitude of force by the cross‐sectional area (0 h *N* = 10; 24 h PL *N* = 11; 24 h OL *N* = 10; 48 h PL *N* = 16; 48 h OL *N* = 16). (D–F) Contractility kinetics of rat LMS (0 h *N* = 8; 24 h PL *N* = 11; 24 h OL *N* = 8; 48 h PL *N* = 16; 48 h OL *N* = 16). (D) Time (in seconds) required to reach peak amplitude of force. (E) Time (in seconds) for the amplitude to decay from maximum force to 50%. (F) Time (in seconds) for the amplitude to decay from maximum force to 90%. (G–H) Functional remodelling of rat LMS after 48 h of IL‐11 treatment. (G) Contractility of LMS as measured by normalising the amplitude of force by the cross‐sectional area (−IL‐11 PL *N* = 8; −IL‐11 OL *N* = 7; +IL‐11 PL *N* = 13; +IL‐11 OL *N* = 12). (H) Time (in seconds) required to reach peak amplitude of force. (I) Time (in seconds) for the amplitude to decay from maximum force to 50%. (J) Time (in seconds) for the amplitude to decay from maximum force to 90% (−IL‐11 PL *N* = 8; −IL‐11 OL *N* = 7; +IL‐11 PL *N* = 7; +IL‐11 OL *N* = 7). Each point represents an individual slice. *P* value was calculated using two‐way ANOVA with Bonferroni's multiple comparison. *, **, ***, **** = *P* value < 0.05, 0.01, 0.001, and 0.0001, respectively. Data shown as mean ± SEM. IL‐11 = interleukin 11. PL, physiological load; OL, overload.

To determine the effect of mechanical overload at a functional level, contractility experiments were conducted using a force transducer at Day 0 and after 24 and 48 h of culture. Maximum contractility (maximum force generation induced by progressive diastolic stretch and normalized to LMS cross‐sectional area) was significantly reduced after 48 h of culture in the overloaded group (*Figure* *3C*). Nevertheless, the application of mechanical overload had no significant effect on contractility kinetics (time to peak and time to 50 and 90% decay) (*Figure* *3D–F*). When the LMS were cultured in the presence of IL‐11, a similar reduction in contractility was only observed at physiological load, showing no differences vs. overloaded LMS (*Figure* *3G*). No differences in contractility kinetics were observed after treatment with IL‐11 (*Figure* *3H–J*).

### Assessing mechanosensitive molecular mechanisms of fibrosis in human living myocardial slices

**Figure 4:**
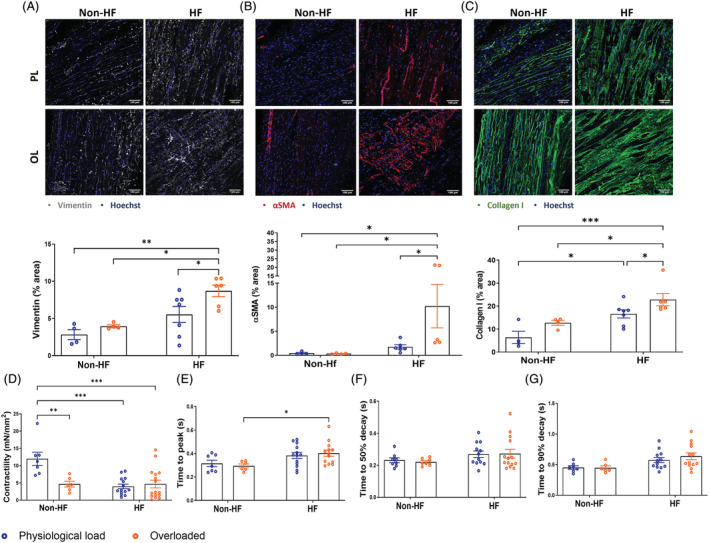
Human living myocardial slices (LMS) for the study of mechanosensitive molecular mechanisms of myocardial fibrosis. (A) Representative images and quantifications of human LMS after 48 h culture stained for Vimentin/Hoechst; (B) αSMA/Hoechst (C) Collagen I/Hoechst (non‐HF PL *N* = 4; non‐HF OL *N* = 4; HF PL *N* = 7; HF OL *N* = 6). (D) Contractility of LMS as measured by normalising the amplitude of force by the cross‐sectional area. (E) Time (in seconds) required to reach peak amplitude of force. (F) Time (in seconds) for the amplitude to decay from maximum force to 50%. (G) Time (in seconds) for the amplitude to decay from maximum force to 90% (non‐HF PL *N* = 4; non‐HF OL *N* = 4; HF PL *N* = 7; HF OL *N* = 6). Each point represents an individual slice. *P* value was calculated using two‐way ANOVA with Bonferroni's multiple comparison. *, **, *** = *P* value <0.05, 0.01, and 0.001, respectively. Data shown as mean ± SEM. HF, heart failure; PL, physiological load; OL, overload.

The LMS were also obtained from the left ventricle of human explanted hearts (dilated cardiomyopathy), and from non‐used donor hearts (*Figures* *4* and *S2*).

We assessed CFs and myofibroblast cell density by immunofluorescence staining before and after 48 h of culture at 2.2 μm SL and 2.4 μm SL. Vimentin‐positive cells, including all stromal cell types, were more numerous in HF than in Non‐HF samples (*Figure* *4A*). Furthermore, the density of αSMA‐positive cells (*Figure* *4B*) and the Collagen I protein level (*Figure* *4C*) was also higher in HF than in non‐HF.

Comparing mechanical loads, in Non‐HF samples there were no significant differences between loads in Vimentin or αSMA protein expression, where the signal mainly came from the microvasculature. However, in HF samples, overloaded LMS had significantly increased the expression of both markers. Collagen I deposition was increased in mechanical overloaded LMS from HF samples.

At a functional level, contractility experiments were conducted after 48 h. Non‐HF LMS cultured at physiological load had the best‐preserved contractility compared with non‐HF overloaded LMS and HF LMS (*Figure* *4D*). There were no observed differences between mechanical load conditions in HF samples. However, in terms of contractility kinetics, time to peak was increased in overloaded LMS from HF samples vs. non‐HF slices (*Figure* *4E–G*).

### Anti‐fibrotic drug testing on living myocardial slices cultured under pathological mechanical load

**Figure 5:**
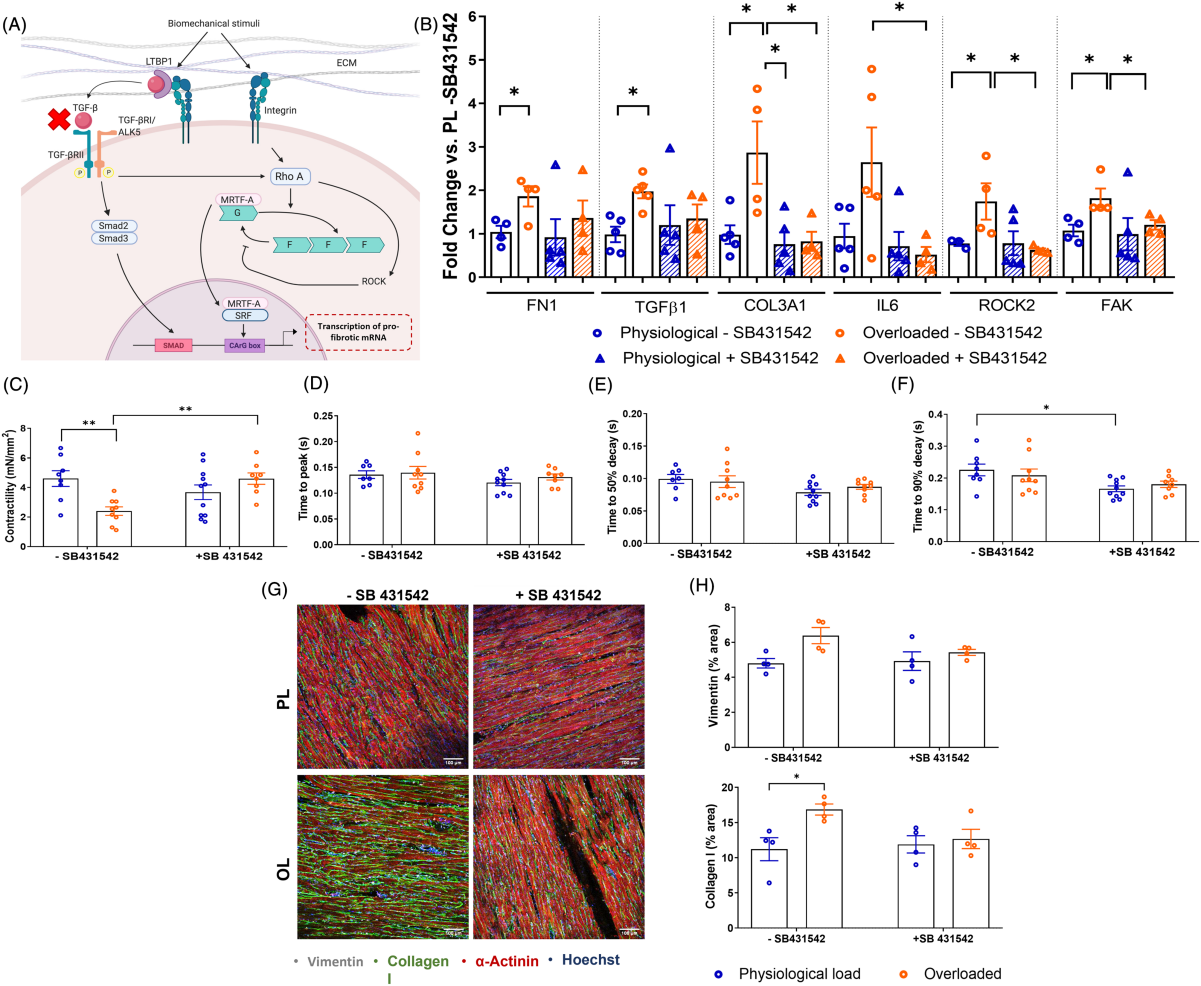
Anti‐fibrotic drug testing on living myocardial slices (LMS) cultured under physiological and pathological mechanical load. (A) TGFβ activation pathway. (B) Fold change expression of 48 h cultured rat LMS (− SB431542 PL *N* = 4–5; − SB431542 OL *N* = 4–5; +SB431542 PL *N* = 5; +SB431542 OL *N* = 4), TGF‐β with and without SB‐431542 treatment of pro‐fibrotic genes FN1, COL3A1, pro‐inflammatory gene IL‐6, genes activated by mechanical stress, ROCK2 and FAK. (C–F) Effects of SB‐431542 on contractility parameters of 48 h cultured rat LMS (−SB431542 PL *N* = 7–8; −SB431542 OL *N* = 9; +SB431542 PL *N* = 10; +SB431542 OL *N* = 7). (C) Contractility of LMS after 48 h culture determined by normalising the amplitude of force by the LMS cross‐sectional area. (D) Time (in seconds) required to reach peak amplitude of force. (E) Time (in seconds) for the amplitude to decay from maximum force to 50%. (F) Time (in seconds) for the amplitude to decay from maximum force to 90%. (G–H) Representative images and quantifications of rat LMS stained for Vimentin, Collagen I, α‐actinin and Hoechst after 48 h of culture with and without SB‐431542 (−SB431542 PL *N* = 4; −SB431542 OL *N* = 4; +SB431542 PL *N* = 4; +SB431542 OL *N* = 4). *P* value was calculated using two‐way ANOVA with Bonferroni's multiple comparison. *, ** = *P* value <0.05 and 0.01, respectively. Data shown as mean ± SEM. SL, sarcomere length; TGF‐βR, transforming growth factor β receptor. PL, physiological load; OL, overload.

TGF‐β1 is a major pro‐fibrotic cytokine. Upon binding to the TGF‐β receptor (TGF‐βR) on fibroblasts, it triggers a signalling cascade which leads to the transcriptional up‐regulation of genes crucial to myofibroblast humoral and biomechanical activation (*Figure* *5A*). Rat LMS were cultured at different mechanical loads with or without 3 μM SB‐431542, a TGF‐βR blocker. Following 48 h, each LMS was assessed for gene expression and functional behaviour.

To determine whether inhibition of TGF‐β1 signalling reduced load‐induced fibrosis, the relative expression of fibrotic and inflammatory markers was assessed by qRT‐PCR. SB‐431542 significantly reduced the difference in FN1 expression between loads. As signal transduction via TGF‐β1 includes multiple complex feedback loops, it was necessary to determine whether blocking the TGFβR resulted in a subsequent increase in TGF‐β1 production that might outweigh the positive effects of the drug. TGF‐β1 expression was greatest in the overloaded condition within the control, but with the addition of SB‐431542, no significant difference between loads was observed. COL3A1 and IL‐6 expression was reduced most in overloaded LMS when treated with SB‐431542. Finally, SB‐431542 down‐regulated the expression of ROCK2 and FAK and reduced the differences between loads, also demonstrating an effect on this mechanosensory pathway (*Figure* *5B*).

Measurement of cardiac contractility is necessary in preclinical safety assessment of therapeutic agents as drug‐induced negative inotropic effects can limit the use of a potential therapy. We measured the acute and the chronic (after culture) effect of SB‐431542 on contractility parameters. To determine the acute inotropic effect of SB‐431542, fresh LMS were stretched to physiological preload and contractility was measured with increasing concentrations of the drug (*Figure S3*). Increasing concentrations of SB‐431542 caused an initial positive inotropic response at 0.5 and 1 μM. However, this was not significant compared with 0 μM and diminished with higher doses. Time to peak did not mirror the change in contractility but was significantly decreased beyond 1 μM by approximately 10%. Concentrations greater than 1 μM also produced a decrease in time to 50% decay demonstrating a positive lusitropic effect.

In control conditions LMS cultured at 2.2 μm SL following 48 h culture (*Figure* *5C*) exhibited higher force of contraction compared with the overloaded LMS. However, when LMS were cultured with SB‐431542, the force of contraction in the overloaded slices was increased. SB‐431542 also had a significant effect on contractility kinetics when cultured with the normally loaded slices, showing a faster time to 90% decay (*Figure* *5D–F*).

Finally, we assessed the effect of SB‐431542 on CFs density and Collagen I protein expression in the ECM after treatment (*Figure* *5G–H*). Protein expression of Collagen I in the ECM was higher in the overloaded LMS, whereas it was reduced with the presence of SB‐431542. There were no differences in Vimentin expression, corresponding to no changes in CFs proliferation.

## Discussion

The *in vitro* exploration of mechanical load and fibrosis has been considerably hampered by the lack of appropriate experimental models that successfully imitate the in vivo process.^4^, ^14^ Here, we set out to test the use of LMS for the study of load‐induced fibrosis. Our results show that overload triggers an up‐regulation of fibrotic, inflammatory and mechanotransduction genes together with an increase in collagen deposition and ECM remodelling in LMS. Furthermore, LMS cultured at pathological load display lower contractility without loss of viability, showing remarkable plasticity to preload changes.

After 48 h, LMS cultured in overloaded conditions showed an increase in nuclear translocation of YAP and up‐regulation of genes activated by mechanical stress (ROCK2, FAK). YAP and TAZ have been identified as sensors and mediators of mechanical cues instructed by the cellular microenvironment.^18^ In fibroblasts, ECM stiffness mechanoactivates YAP/TAZ nuclear translocation which promotes the production of profibrotic mediators and ECM proteins.^18^ By contrast, FAK is activated by integrins in response to mechanical stress, and signals downstream to activate mitogen‐activated protein kinase signalling, inducing myofibroblast differentiation and ECM production.^19^, ^20^

The multicellularity, 3D nature and presence of viable myocardium in LMS in culture permit CFs to maintain physiological interactions with other cardiac cells and the ECM.^12^, ^15^ The gene expression pattern in isolated CFs from cultured LMS showed up‐regulation of mechanical stress‐related genes in overloaded conditions and, at the same time, an increase in their fibrotic phenotype and in myofibroblast differentiation, suggesting the fibrotic effect of mechanical overload to this cell type.

In order to compare load‐depended and load‐independent events, the pro‐fibrotic protein IL‐11 was added to the culture media. TGF‐β1 is the principal pro‐fibrotic factor, but its inhibition is associated with side effects due to its pleiotropic roles.^21^ The up‐regulation of IL‐11 is the dominant transcriptional response to TGF‐β1 exposure and is required for its pro‐fibrotic effect. IL‐11 and its receptor (IL‐11RA) are expressed specifically in CFs, in which they drive a non‐canonical, ERK‐dependent autocrine signalling pathway required for fibrogenic protein synthesis. It is known that inhibition of IL‐11 prevents fibroblast activation across organs and species in response to a range of important pro‐fibrotic stimuli.^16^ We found that IL‐11 activated fibrogenic protein synthesis, including collagen deposition and expression, αSMA expression and fibroblast proliferation in LMS cultured at physiological load but not in overstretched LMS. Functionally, with the presence of IL‐11 in the media, a reduction in contractility was observed in LMS stretched at physiological load. These results corroborate a load‐independent effect of IL‐11 and indicate that the application of a continuous pathological mechanical load could induce similar functional and remodelling profibrotic effects than those observed with the addition of IL‐11.

Human cardiac tissue is of great value for translational studies. LMS were also successfully prepared from tissue samples obtained from explanted HF hearts and non‐HF hearts. HF LMS demonstrated a reduction in contraction force and an increased fibrosis vs. non‐HF slices. Interestingly, non‐HF LMS had a similar terminal phenotype to healthy rat myocardial slices. Overloading healthy LMS induced a significant reduction in contractility, reaching similar values to HF‐LMS. However, there were no differences in fibroblasts proliferation and no differences in αSMA positive cells. Collagen production was increased but not significantly. The outcome is different when pathological LMS are used. Overloading HF LMS significantly increased fibroblasts proliferation, collagen deposition and the number of αSMA positive fibroblasts, showing a more advanced fibrotic response. In terms of functionality, no differences were found between loads in HF LMS, which can be attributed to pre‐existing tissue damage. These findings support the effect of mechanical load at functional level but indicate that in both, human and animal healthy LMS the time of culture would have to be increased to observe an advanced fibrotic response, including the presence of myofibroblasts expressing αSMA.

Differences between Non‐HF and HF can also be explained considering the process of tissue remodelling and the heterogeneous population of CFs in the LV of the failing heart.^22^, ^23^ In the healthy myocardium, quiescent CFs are protected from large changes in mechanical load by their physical integration in a structurally stable ECM. Nevertheless, CFs from a failing myocardium are less protected against mechanical stress due to rapid degradation and loss of native ECM stiffness and this is associated with CFs proliferation and production of matricellular and structural ECM proteins that increase the stiffness of the tissue.^24^

Load‐induced myofibroblast differentiation permits rapid activation of TGF‐β1, amplifying the fibrotic response.^25^, ^26^ Some experimental studies showed that modulation of TGF‐β1 signalling reduced interstitial fibrosis and improved left ventricular function.^22^, ^27^ Here, we observed that overloaded LMS had increased expression of TGF‐β1. Therefore, we used an inhibitor of the TGF‐β1 pathway to prevent the fibrotic phenotype after mechanical overload in our LMS model. SB‐431542, a TGF‐β1R blocker, produced a significant decrease in profibrotic gene expression and Collagen I production in overloaded conditions. Similar effects were previously reported in cultured neonatal rat CFs; α‐SMA and Collagen I expression was suppressed by SB‐431542 via SMAD‐2 and p38 signalling pathways.^26^ IL‐6 expression was also reduced in the overloaded LMS, thus imparting an anti‐inflammatory effect. Although this contradicts reports that TGF‐β1 behaves as an anti‐inflammatory cytokine in vivo which was deemed beneficial post‐MI,^28^, ^29^ TGF‐β1 is highly pleiotropic and its immunomodulatory effect is dependent on the environment it is in. Thus, with increased preload, TGF‐β1 may induce inflammation explaining the anti‐inflammatory effect observed here when using SB‐431542. Genes associated with mechanical stress were down‐regulated with the addition of the TGF‐βR blocker, reinforcing the major role that TGF‐β1 plays in load‐induced remodelling.

An anti‐fibrotic drug must also result in improved cardiac function to produce a clinical benefit. The TGF‐βR blocker successfully prevented a significant deterioration in contractility of LMS cultured under mechanical overload suggesting that inhibiting the TGF‐β pathway may be beneficial *in vivo*. Nevertheless, the effect of anti‐TGF‐β therapy on cardiac function *in vivo* has been controversial. Abnormal diastolic dilation leading to systolic failure and death has been shown before.^30^ A possible explanation for this discrepancy is the length and timing of treatment. We showed no adverse effects on contractility in LMS cultured for 48 h, but we cannot exclude pejorative effects after longer periods. Promising therapeutic targets could be signalling factors downstream from TGF‐β1. In mice with pressure‐overload–induced interstitial fibrosis, overexpression of CCN5 interfered with TGF‐β1 signalling, enhanced myofibroblast apoptosis, and reduced established fibrosis.^31^ Another possible strategy could be to target more than one pathway for treatment of fibrosis combining TGF‐β1 signalling path blockers and cardiac unloading.^32^

In conclusion, using a platform, which responds with myocardial remodelling differently to physiological and pathological load, we demonstrated an up‐regulation of mechanotransduction genes, ECM remodelling, an increase of the fibrotic phenotype, and a decrease in functionality under mechanical overloaded conditions. Moreover, the effect of mechanical overload was comparable with the effect of the profibrotic protein IL‐11. Finally, blocking the TGF‐βR pathway demonstrated the remarkable ability to prevent overload‐induced fibrosis and inflammation. These findings support the use of LMS for the study of myocardial fibrosis associated with relevant changes in mechanical load and may lead to the development of novel therapeutic strategies for cardiac disease.

## Conflict of interest

Non declared.

## Funding

This work was supported by the British Heart Foundation (project grants PG/17/61/33187, PG/20/8/34856 to R.N.T and C.M.T).

## Supporting information


**Figure S1.** **Cardiac Fibroblasts isolation.** (a) LMS were digested immediately after culture with collagenase‐A. Rat Cardiac Fibroblast Isolation Kit (Milteny Biotec, UK) and a MACS separator were used to separate cardiac fibroblasts from non‐cardiac fibroblasts. (b) Immunostaining of non‐fibroblasts markers (CD31, CD45) and fibroblasts markers (Vimentin, Fibroblast Specific Protein; FSP‐1) was performed to confirm the cell isolation methods (Supplementary Figure 1b).
**Figure S2. Human LMS slices before culture.** (a) Representative images and (b) quantifications of human LMS before culture stained for Vimentin/Hoechst; (b) αSMA/Hoechst (c) Collagen I/Hoechst (Non‐HF *N* = 4; HF *N* = 6). *P*‐value was calculated using a Student's t test. * = *p* value < 0.05. Data shown as mean ± SEM. HF = Heart Failure.
**Figure S3. Fold change in contractility parameters with increasing doses of TGF‐β blocker, SB‐431542**. (c) Contractility of LMS as measured by normalising the amplitude of force by the cross‐sectional area. (d) Time (in seconds) required to reach peak amplitude of force. (e) Time (in seconds) for the amplitude to decay from maximum force to 50%. (f) Time (in seconds) for the amplitude to decay from maximum force to 90%. (*N* = 4/2).
**Table S1. Patient characteristics.** DCM = dilated cardiomyopathy.
**Table S2.** **List of primers used for cDNA amplification by qPCR.**
