# Supplementary material for: Mechanosensitive molecular mechanisms of myocardial fibrosis in living myocardial slices
Source: ESC Heart Fail. 2022 Feb 6;9(2):1400–12. doi: 10.1002/ehf2.13832 (PMC8934971; doi:10.1002/ehf2.13832)
Supplement: Supplementary file 1 — Figure S1. Cardiac Fibroblasts isolation. (a) LMS were digested immediately after culture with collagenase‐A. Rat Cardiac Fibroblast Isolation Kit (Milteny Biotec, UK) and a MACS separator were used to separate cardiac fibroblasts from non‐cardiac fibroblasts. (b) Immunostaining of non‐fibroblasts markers (CD31, CD45) and fibroblasts markers (Vimentin, Fibroblast Specific Protein; FSP‐1) was performed to confirm the cell isolation methods (Supplementary Figure 1b). Figure S2. Human LMS slices before culture. (a) Representative images and (b) quantifications of human LMS before culture stained for Vimentin/Hoechst; (b) αSMA/Hoechst (c) Collagen I/Hoechst (Non‐HF N = 4; HF N = 6). P‐value was calculated using a Student's t test. * = p value < 0.05. Data shown as mean ± SEM. HF = Heart Failure. Figure S3. Fold change in contractility parameters with increasing doses of TGF‐β blocker, SB‐431542. (c) Contractility of LMS as measured by normalising the amplitude of force by the cross‐sectional area. (d) Time (in seconds) required to reach peak amplitude of force. (e) Time (in seconds) for the amplitude to decay from maximum force to 50%. (f) Time (in seconds) for the amplitude to decay from maximum force to 90%. (N = 4/2). Table S1. Patient characteristics. DCM = dilated cardiomyopathy. Table S2. List of primers used for cDNA amplification by qPCR. [file EHF2-9-1400-s001.pdf]

Mechanosensitive molecular mechanisms of myocardial fibrosis in Living Myocardial Slices

Raquel Nunez-Toldra, Thomas Kirwin[1], Elisa Ferraro, Fotios G. Pitoulis, Laura Nicastro, Ifigeneia Bardi, Worrapong Kit-Anan, Julia Gorelik, Andre R. Simon & Cesare M. Terracciano

APPENDICES

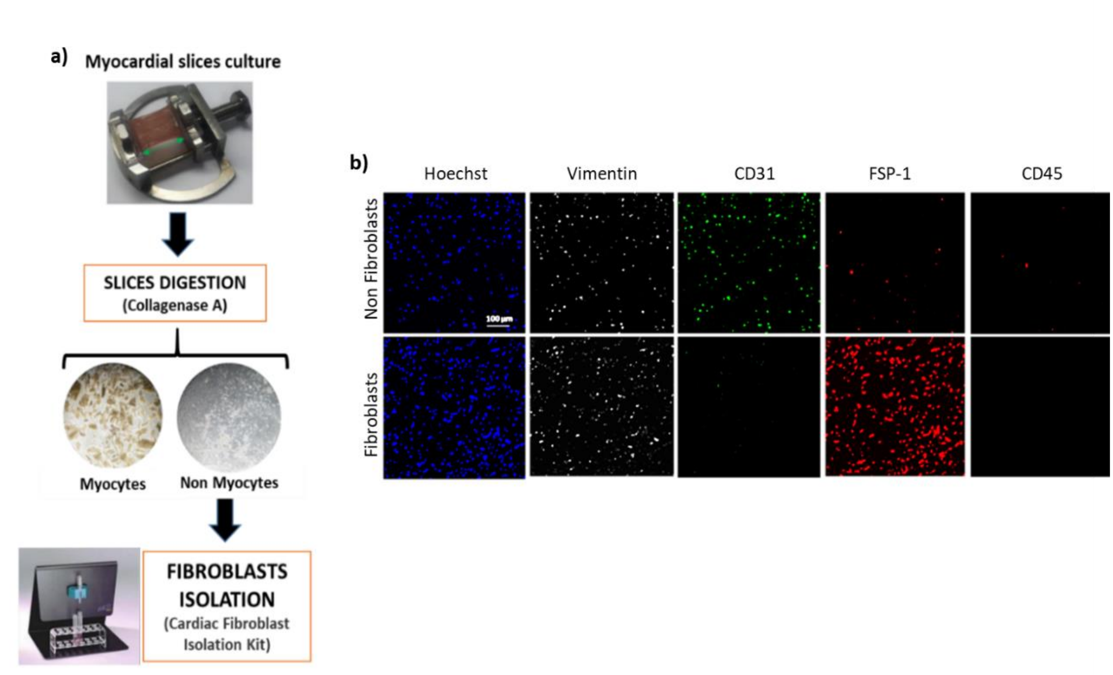

Fig. S1. Cardiac Fibroblasts isolation. (a) LMS were digested immediately after culture with collagenase-A. Rat Cardiac Fibroblast Isolation Kit (Milteny Biotec, UK) and a MACS separator were used to separate cardiac fibroblasts from non-cardiac fibroblasts. (b) Immunostaining of non-fibroblasts markers (CD31, CD45) and fibroblasts markers (Vimentin, Fibroblast Specific Protein; FSP-1) was performed to confirm the cell isolation methods (Supplementary Figure 1b).

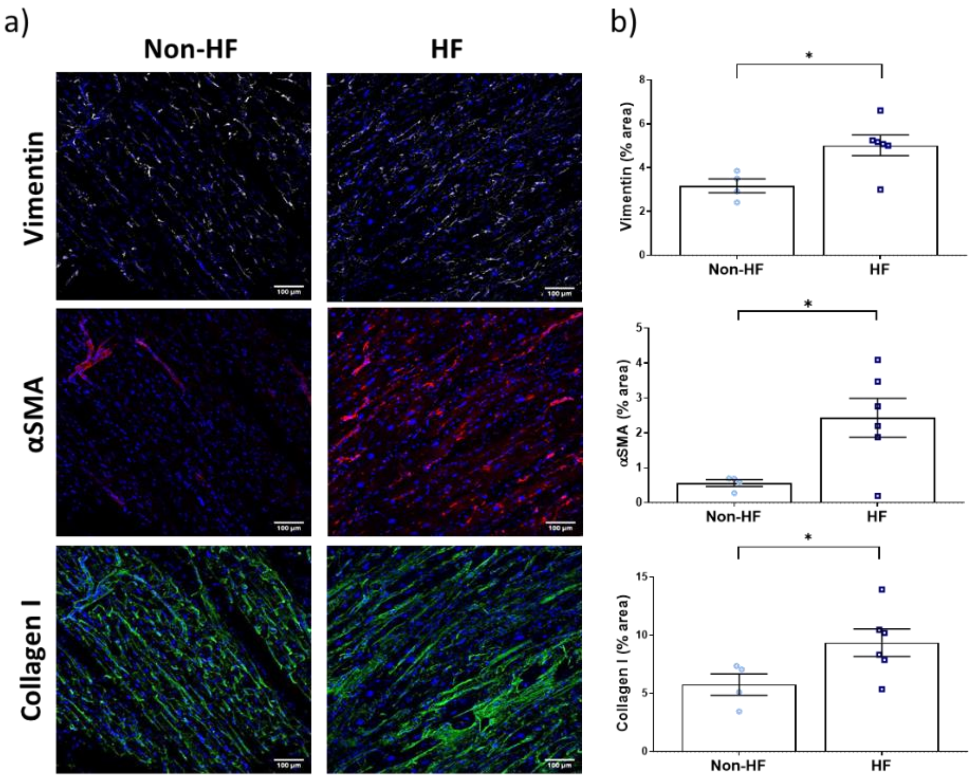

Fig. S2. Human LMS slices before culture. (a) Representative images and (b) quantifications of human LMS before culture stained for Vimentin/Hoechst; (b) αSMA/Hoechst (c) Collagen I /Hoechst (Non-HF N=4; HF N=6). P-value was calculated using a Student's t test. * = p value < 0.05. Data shown as mean ± SEM. HF= Heart Failure.

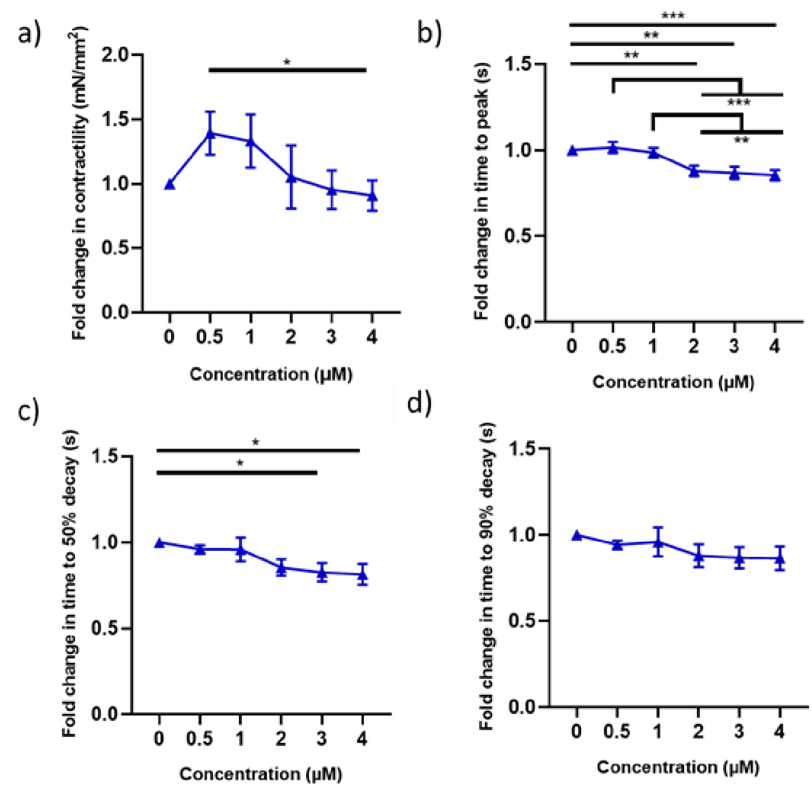

Fig. S3. Fold change in contractility parameters with increasing doses of TGF-β blocker, SB-431542. (c) Contractility of LMS as measured by normalising the amplitude of force by the cross-sectional area. (d) Time (in seconds) required to reach peak amplitude of force. (e) Time (in seconds) for the amplitude to decay from maximum force to 50%. (f) Time (in seconds) for the amplitude to decay from maximum force to 90%. (N=4/2).

Sample	Diagnosis	Age (years)	Gender
Non-HF 1	N/A	50	Female
Non-HF 2	N/A	17	Male
Non- HF 3	N/A	51	Male
Non-HF 4	N/A	40	Male
HF 1	DCM	56	Male
HF 2	DCM	35	Male
HF 3	DCM	63	Female
HF 4	DCM	59	Male
HF 5	DCM	45	Male
HF 6	DCM	21	Male
HF 7	DCM	64	Male

Table S1. Patient characteristics. DCM= dilated cardiomyopathy.

Gene	Forward Primer (5’-3’)	Reverse Primer (5’-3’)
GAPDH	AGTTCAACGGCACAGTCAAG	TACTCAGCACCAGCATCACC
ACTA2	CATCACCAACTGGGACGACA	TCCGTTAGCAAGGTCGGATG
FN1	GGATCCCCTCCCAGAGAAGT	GGGTGTGGAAGGGTAACCAG
TGFβ-1	CCTGGAAAGGGCTCAACAC	CAGTTCTTCTCTGTGGAGCTGA
COL3A1	CCACCCTGAACTCAAGAGCG	ACAGTCATGGGACTGGCATT
IL-6	TCTCTCCGCAAGAGACTTCCA	ATACTGGTCTGTTGTGGGTGG
SMAD3	ATCCGCATGAGCTTCGTCAA	TCCCCAACCCGATCCCTTTA
SMAD4	ACGCCGTCTTCGTGCAGAG	TGACACTGCCGCAAATCAAAG
ROCK2	CCCGATCATCCCCTAGAACC	TTGGAGCAAGCTGTCGACTG
FAK	AACAATGCGCCAGTTTGACC	CTCTCCAGATACGCGAGTGC

Table S2. List of primers used for cDNA amplification by qPCR.
